# *Ocimum gratissimum* Aqueous Extract Induces Apoptotic Signalling in Lung Adenocarcinoma Cell A549

**DOI:** 10.1155/2011/739093

**Published:** 2010-09-26

**Authors:** Han-Min Chen, Mu-Jang Lee, Cheng-Yi Kuo, Pei-Lin Tsai, Jer-Yuh Liu, Shao-Hsuan Kao

**Affiliations:** ^1^Department of Life Science, Fu-Jen Catholic University, Taipei 24205, Taiwan; ^2^Department of Internal Medicine, Division of Cardiology, Tian-Sheng Memorial Hospital, Pingtung 92843, Taiwan; ^3^Multi Chemical Laboratory, SGS Taiwan Ltd., Taipei 24803, Taiwan; ^4^Institute of Biochemistry and Biotechnology, Chung Shan Medical University, Taichung City 40201, Taiwan; ^5^Graduate Institute of Cancer Biology, China Medical University and Hospital, Taichung City 40402, Taiwan; ^6^Clinical Laboratory, Chung Shan Medical University Hospital, Taichung City 40201, Taiwan; ^7^Institutes of Biochemistry and Biotechnology, Chung Shan Medical University, No. 110, Sec. 1, Jianguo N. Road, Taichung 40201, Taiwan

## Abstract

*Ocimum gratissimum* (OG) is widely used as a traditional herb for its antibacterial activity in Taiwan. Recently, antitumor effect of OG on breast cancer cell is also reported; however, the effects of OG on human pulmonary adenocarcinoma cell A549 remain unclear. Therefore, we aimed to investigate whether aqueous OG extract (OGE) affects viability of A549 cells and the signals induced by OGE in A549 cells. Cell viability assays revealed that OGE significantly and dose-dependently decreased the viability of A549 cell but not that of BEAS-2B cell. Morphological examination and DAPI staining indicated that OGE induced cell shrinkage and DNA condensation for A549 cells. Further investigation showed that OGE enhanced activation of caspase-3, caspase-9 and caspase-8 and increased protein level of Apaf-1 and Bak, but diminished the level of Bcl-2. Additionally, OGE inhibited the phosphorylation of extracellular signal-regulated kinase (ERK) yet enhanced the phosphorylation of c-Jun N-terminal kinase (JNK) and p38 MAP kinase (p38). In conclusion, our findings indicate that OGE suppressed the cell viability of A549 cells, which may result from the activation of apoptotic signaling and the inhibition of anti-apoptotic signaling, suggesting that OGE might be beneficial to lung carcinoma treatment.

## 1. Introduction

Lung adenocarcinoma is the major cause of cancer-related mortality worldwide [1, 2]. Despite clinical success of concurrent therapeutic approaches against lung cancer including chemotherapy and radiotherapy, marked chances of undesirable and adverse side effects caused by such therapies need to be managed. Use of medicinal plants in chemoprevention is considered as an ideal treatment with good efficacy and few side effects compared with allopathic medicine [3]. Mounting evidences have shown that dietary intake of phytochemicals, an important group of chemopreventive agents, reduces the risk of cancer [4] and has antitumor potential against lung cancer [5]. 

Chemopreventive agents are compounds that prevent development of cancer. Their preventive effects are attributed to [1] intervening in interaction of the carcinogen with cellular DNA, [2] altering intracellular signaling pathways as results of stopping progression of an initiated cell through preneoplastic changes into a malignant cell, [3] inhibiting angiogenesis, [4] inducing cell cycle arrest, and [5] triggering apoptosis. It is believed that the apoptosis induced by chemopreventive agents may not only inhibit the carcinogenesis induced by carcinogens, but also may suppress the growth of tumor and enhance the cytotoxic effects of antitumor drug on tumor, which plays a pivotal role in the antitumor therapies [6].

The genus *Ocimum*, belonging to the family *Labiatae*, is widely found in tropical and subtropical regions. The widespread plant is known for its chemopreventive, anticarcinogenic, free radical scavenging, and other pharmacological properties and used as a traditional herb in European and Asian countries since ancient times [7]. It has been prepared in a variety of forms for consumption. The aqueous leaf extract and seed oil are reported to show chemopreventive and antiproliferative activity on Hela cells [8]. Ethanolic extract of *Ocimum* leaf also has been shown to have significant modulatory influence on carcinogen metabolizing enzymes including cytochrome P450, cytochrome b5, and aryl hydrocarbon hydroxylase, glutathione-S-transferase [9, 10]. Additionally, *Ocimum sanctum* prepared in the form of fresh leaf paste, aqueous, and ethanolic extract has been reported to reduce the incidence of papillomas and squamous cell carcinoma in carcinogen-treated hamsters with an observation that the aqueous extract exerts more profound effect than the other two forms [11]. Nevertheless, the mechanisms of aqueous extract of *Ocimum gratissimum* (OGE) underlying its anticancer property remain sketchy.

In the present study, the anticancer effects of OGE were investigated using human lung carcinoma A549 cells. The effects of OGE on cell viability and apoptosis of A549 cell were determined by measuring the activity of mitochondrial malate dehydrogenase and the DNA condensation, respectively. The OGE-induced caspase activation cascades and kinase signaling, including caspase-3, caspase-8, caspase-9, apaf-1, Bcl-2, Bak, extracellular signal-regulated kinase (ERK), Akt, c-Jun N-terminal kinase (JNK), and p38 mitogen-activated protein (MAP) kinase (p38), were elucidated using immunological approaches

## 2. Materials and Methods

### 2.1. Materials

[3-(4,5-dimethylthiazol-2-yl)-2,5-diphenyl-tetrazolium bromide (MTT), 4′-6-Diamidino-2-phenylindole (DAPI), penicillin, and streptomycin were purchased from Sigma (St. Louis, MO). 

Dulbecco's modified Eagle's medium (DMEM), fetal bovine serum (FBS), and trypsin-EDTA were purchase from Gibco BRL (Gaithersburg, MD). Antibodies against caspase-3, caspase-8, caspase-9, apaf-1, Bak, Bcl-2, phosphorylated-ERK1/2 (p-ERK1/2), ERK1/2, phosphorylated-JNK (p-JNK), JNK, phosphorylated-p38 (p-p38), and p38 were purchased from Cell Signaling Technologies (Beverly, MA). Antibodies against *β*  actin and Glyceraldehyde 3-phosphate dehydrogenase (GAPDH) were obtained from Sigma. HRP-conjugated secondary antibodies against mouse IgG and rabbit IgG were purchased from Abcam Inc. (Cambridge, UK). The lung adenocarcinoma cell A549 was obtained from American Type Culture Collection (ATCC; Rockville, MD).

### 2.2. Preparation of OGE and Composition Analysis

Leaves of *Ocimum gratissimum* Linn were harvested and washed with distilled water followed by homogenization with distilled water using polytron. The homogenate was incubated at 95°C for 1 hour (h) and then filtered through two layers of gauze. The filtrate was centrifuged at 20,000 g, 4°C for 15 minute to remove insoluble pellets and the supernatant (OGE) was thereafter collected, lyophilized, and stored at −70°C until use.

The content of polyphenol in OGE was analyzed as previously described in [12] and revealed the final extract (OGE) contained 11.1% polyphenolic acid and 4.5% flavonoids.

### 2.3. Cell Culture and Experimental Treatments

Lung adenocarcinoma cell A549 and SV40-transformed normal epithelial cell BEAS-2B were maintained in DMEM supplemented with 10% FBS and 100 *μ*g/ml penicillin/streptomycin at 37°C in a humidified atmosphere containing 5% CO_2_. Cells were seeded in 6-well culture plates at an initial density of 1 × 10^5^ cells/ml and grown to approximately 80% confluence. OGE treatments were performed by incubating cells with serial concentrations of OGE (w/v) in serum-free DMEM for 48 h. After the OGE treatments, the cells were washed with phosphate-buffered saline (PBS; 25 mM sodium phosphate, 150 mM NaCl, pH 7.2) and collected for following analyses.

### 2.4. DAPI Staining

DAPI staining was performed to assess morphological changes in the chromatin structure of A549 cells undergoing apoptosis as previously described in [13]. Briefly, cells were trypsinized, mounted on glass slides, and fixed in 4% paraformaldehyde for 30 minutes. followed by staining with 1 *μ*g/ml DAPI for 30 minutes Apoptosis was characterized by chromatin condensation and fragmentation when examined by fluorescence microscopy. The incidence of apoptosis in each treatment was analyzed by counting 300 cells and presented in the percentage of apoptotic cells.

### 2.5. Cell Viability Assay

Cell viability was determined by MTT assay [14] in the absence or presence of 50 or 100 *μ*g/ml OGE. After 48 h treatments, culture medium was aspirated and cells were incubated with MTT (0.5 mg/ml) at 37°C for 4 h. The viable cell number was directly proportional to the production of formazan, which was dissolved in isopropanol and determined by measuring the absorbance at 570 nm using a microplate reader (SpectraMAX 360 pc, Molecular Devices, Sunnyvale, CA). 

### 2.6. Immunoblotting

A549 cells were washed with PBS and lysed with lysis buffer (50 mM Tris-HCl, pH 7.5, 150 mM NaCl, 1% Nonidet P-40, 1 mM phenylmethylsulfonyl fluoride, 1 mM sodium fluoride, and 10 *μ*g/ml aprotinin and leupeptin). The lysates were incubated on ice for 30 minutes and centrifuged at 20,000 g for 15 minutes. The supernatants were collected and measured for protein concentration using Bradford method. Crude proteins (30 *μ*g per lane) were subjected to a 12.5% SDS-polyacrylamide gel, and then transferred onto a nitrocellulose membrane (Millipore, Bedford, MA). The blotted membrane was blocked with 5% w/v skimmed milk in PBS, and then incubated for 2 h with 1/1000 dilution of antibodies against human caspase-3, caspase-8, caspase-9, apaf-1, Bak, Bcl-2, p-ERK1/2, ERK1/2, p-JNK, JNK, p-p38, p38, *β*-actin, and GAPDH, respectively. Antigen-antibody complex were detected using 1/2000 dilution of peroxidase-conjugated secondary antibodies and displayed using ECL chemiluminescence reagent (Millipore).

### 2.7. Statistical Analysis

Data were expressed as means ±  SEMs of the three independent experiments. Statistical significance analysis was determined by using 1-way ANOVA followed by Dunnett for multiple comparisons with the control. The differences were considered significant for *P* values less than .05.

## 3. Results

### 3.1. Growth Suppression of Human Lung Carcinoma A549 Cells by OGE Administration

**Figure 1:**
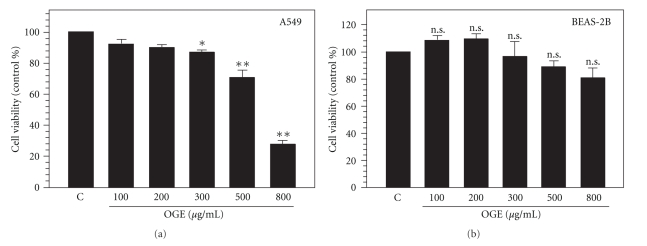
OGE diminished cell viability of A549 cell. The cell viability of A549 cells (a) and BEAS-2B cells (b) treated with serial concentrations of OGE (10, 50, 100, 200 and 300 *μ*g/ml) for 48 h was determined. Data were expressed as mean ±  SEM for 3 independent experiments. *P < .05 and, **P < .01 as comparing to control (c). n.s., no statistical significance.

To examine antitumor activity of OGE, both lung carcinoma cell A549 and epithelial cell BEAS-2B were treated with serial concentrations of OGE (100, 200, 300, 500, and 800 *μ*g/ml) for 48 h, and subsequently analyzed for the cell viability. As shown in Figure 1(a), the viability of A549 cells exposed to OGE for 48 hours was found decreased in association with the concentration of OGE in a dose-dependent fashion. The viability was significantly decreased to 92.3 ± 3.2%, 90.1 ± 1.4%, 87.2 ± 1.2%, 70.6 ± 3.3%, and 27.5 ± 1.4% of control with 100, 200, 300, 500 and 800 *μ*g/ml OGE, respectively (*P* < .05 as compared to control). Interestingly, the cell viability of BEAS-2B was insignificantly affected by OGE treatment. However, the variation of the viability was not statistically significant (.061 < *P* < .121 as compared to control) (Figure 1(b)). Accordingly, the findings showed that 500, and 800 *μ*g/ml OGE treatment significantly suppressed the viability of A549 cells but not that of BEAS-2B.

### 3.2. OGE-Induced Morphological Alteration and DNA Condensation in A549 Cells

**Figure 2:**
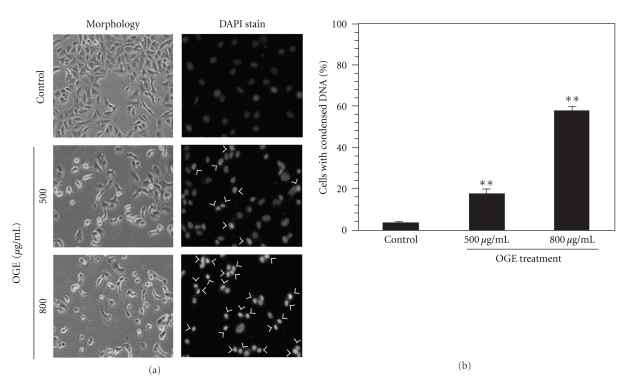
OGE altered cell morphology and induced DNA condensation of A549 cell. A549 cells were treated with 0, 500 and 800 *μ*g/ml OGE for 48 h and then stained with DAPI. (a) The cell morphology and the DNA condensation was photographed by fluorescence microscopy (200x). The cells presented DNA condensation were indicated by arrow. (b) The incidence of DNA condensation was determined for the A549 cells with different treatments. Data were expressed as mean ±  SEM for 3 independent experiments.

To explore the mechanism underlying cell death in presence of OGE, cell morphology and chromatin condensation of OGE-treated A549 cells was examined. As shown in Figure 2(a), OGE treatment altered the morphology of A549 cells as results of cell shrinkage and detachment (left panel). Additionally, a phenomenon of chromatin condensation was observed in the A549 cells treated with OGE in a dose-dependent manner (right panel). The incidence of DNA condensation was quantified, showing that 500 and 800 *μ*g/ml OGE treatment resulted in significant chromatin condensation (17.5 ± 1.7% and 56.8 ± 2.3%, *P* < .01 as compared to control) in A549 cell (Figure 2(b)).

### 3.3. Activation of Both Intrinsic and Extrinsic Apoptotic Pathway in A549 Cells following OGE Treatment

**Figure 3:**
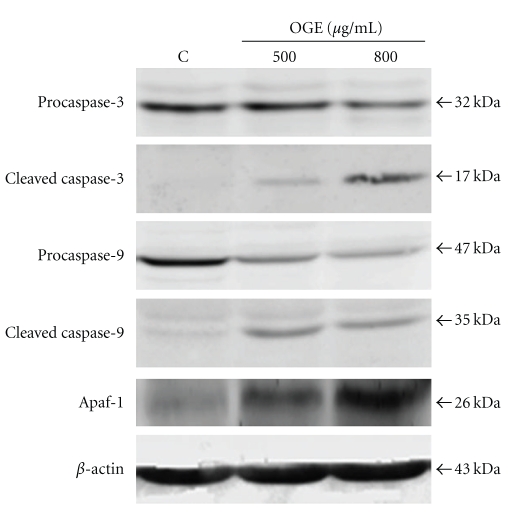
OGE induced activation of intrinsic/mitochondrial apoptotic pathway. A549 cells were treated with 0, 500 and 800 *μ*g/ml for 48 hr, and then were lyzed for the determination of protein levels of caspase-3, cleaved caspase-3, caspase-9, cleaved caspase-9 and Apaf-1 by immunoblotting. *β*-actin was used as control. The apparent molecular weights for detected proteins were indicated.

To discriminate the apoptotic mechanisms induced by OGE treatment, the activations of intrinsic and extrinsic caspase cascades were investigated. As shown in Figure 3, the level of procaspase-3, an important effecter caspase, was decreased in response to OGE treatment in a dose-dependent manner. Concomitant with the decrease of procaspase-3, the increase in cleaved caspase-3 in A549 cells was observed in association with OGE treatment in a dose-dependent fashion. The content of caspase-9 and Apaf-1, two upstream activators of procaspase-3 in intrinsic pathway, were further investigated. The treatment of A549 cells with OGE resulted in the decrease of procaspase-9 and the increase of its cleavage product. The level of Apaf-1 was also dose-dependently increased by OGE treatment (Figure 3). 

**Figure 4:**
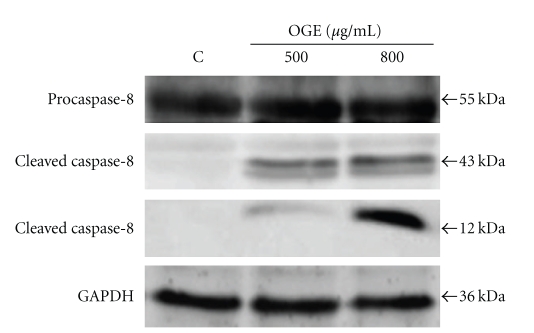
OGE induced activation of caspase-8. A549 cells were treated with 0, 500 and 800 *μ*g/ml for 48 hr, and then were lyzed for the determination of protein levels of caspase-8 and cleaved caspase-8 by immunoblotting. *β*  actin was used as control. The apparent molecular weights for detected proteins were indicated.

The effect of OGE treatment on caspase-8, an upstream activator of caspase-3 in extrinsic pathway, was also investigated. Although no obvious change in the level of procaspase-8 was observed in presence of OGE, the levels of two cleaved caspase-8 (43 kDa and 12 kDa) were remarkably increased by OGE treatment (Figure 4).

### 3.4. Modulation of Bak and Bcl-2 in OGE-Treated A549 Cells

**Figure 5:**
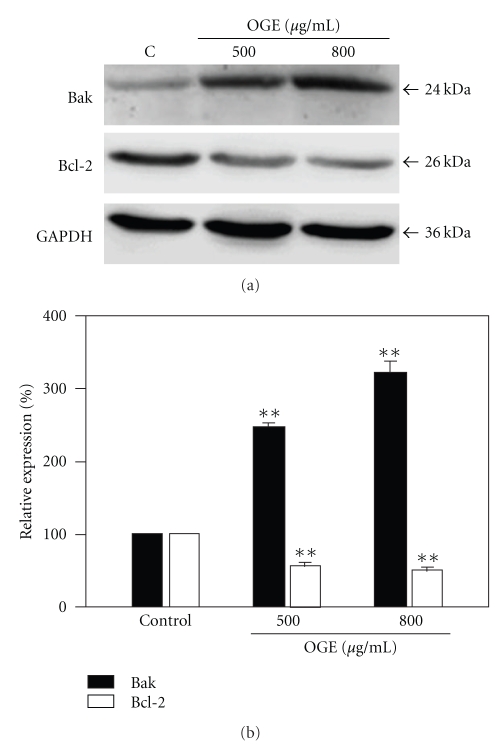
OGE enhanced protein level of Bak and diminished protein level of Bcl-2. A549 cells were treated with 0, 500, and 800 *μ*g/ml for 48 hr, and then were lyzed for the determination of protein levels by immunoblotting. (a) The expression levels of Bak and Bcl-2 were determined. GAPDH was used as control. (b) The expression levels of Bak and Bcl-2 were quantitatively expressed after being standardized to GAPDH. Data are expressed as mean ±  SEM for 3 independent experiments for each concentration point. **P < .01 as compared with control.

To further elucidate the putative mechanism underlying the OGE-associated apoptotic signaling, the levels of proapoptotic proteins Bak and antiapoptotic proteins Bcl-2 at various concentrations of OGE were examined. After being normalized and verified with GAPDH, expression of Bax was increased remarkably in a dose-dependent manner. Moreover, an obvious decrease in the Bcl-2 protein level was detected in the OGE-treated A549 cells as compared to the control (Figure 5(a)). 

Quantitative Bak and Bcl-2 expression after being standardized to GAPDH (*n* = 3) was shown in Figure 5(b). Expression of Bak was increased significantly upon OGE treatment, whereas the significant decrease in expression of Bcl-2 in A549 cells. With 800 *μ*g/ml OGE treatment, the level of Bak and Bcl-2 was increased to 321.3 ± 11.4% and decreased to 50.2 ± 2.2%, respectively (*P* < .01 as compared to control).

### 3.5. Activation of JNK and p38 but Not ERK MAP Kinase in A549 Cells following OGE Treatment

**Figure 6:**
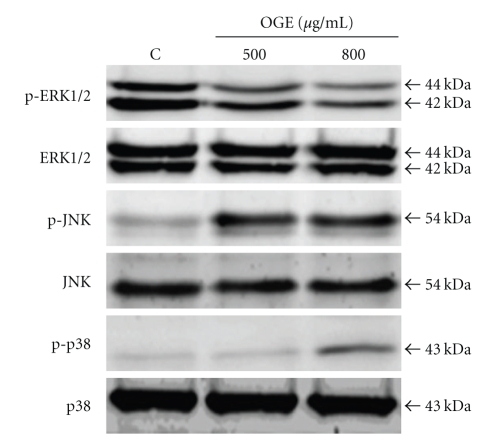
OGE inhibited phosphorylation of Erk but enhanced phosphorylation of JNK and p38. A549 cells were treated with 0, 500 and 800 *μ*g/ml for 48 hr, and then were lyzed for the determination of protein levels by immunoblotting. The levels of p-ERK1/2, ERK1/2, p-JNK, JNK, p-p38, and p38 were presented.

MAP kinases have been widely reported for their involvements in the survival, proliferation, differentiation, and apoptosis in different cancer cells [15]. Therefore, the influence of OGE treatment on activation of three important MAP kinases, ERK, JNK, and p38, were further investigated. As shown in Figure 6, remarkable phosphorylation of ERK, but not JNK and p38, was detected in the control A549 cells. OGE treatment significantly inhibited the phosphorylation of ERK and enhanced the phosphorylation of JNK and p38. 

## 4. Discussion

Aberrant cells such as mutated or proliferating neoplastic cells are removed by programmed cell death, namely, apoptosis [16]. Two well-known pathways, extrinsic and intrinsic pathways, are responsible for triggering apoptosis [17]. In the case of the intrinsic pathway, a release of cytochrome C from mitochondria results in binding to Apaf-1 and subsequently leads to activation of procaspase-9 and following caspase-3 [18]. Activated caspase-3 exerts as the key executioner of apoptosis to induce the cleavage and inactivation of key cellular protein [18, 19]. In the present study, it is demonstrated that OGE treatment increased the Apaf-1 expression level and activated the caspase-9 and 3 cascade. Additionally, it is known that caspase-3 can be activated by caspase-8 through the extrinsic pathway [20]. Our results showed that OGE treatment simultaneously induced the activation of caspase-8. These findings indicate that both activations of the intrinsic and extrinsic pathway are of responses to exposure to OGE in A549 cells as results of apoptosis.

MAP kinase cascades consist of a core of three protein kinases such as ERK1/2, p38, and JNK pathways [21, 22]. Thus, to understand the molecular mechanism of OGE, the potential involvement of MAP kinase pathway in OGE-induced apoptosis was investigated by immunoblotting. The aberrant expressions of Akt and ERK are known as a prominent feature of many human cancers including nonsmall cell lung cancer [23]. Our findings are consistent with the aberration that a relative high level of ERK phosphorylation in the control A549 cells, which may contribute to the malignancy and high frequency of metastasis of lung cancer. Interestingly, our results that ERK phosphorylation in A549 cells was significantly inhibited in presence of OGE suggest that OGE treatment could have suppressive influence on the constitutive survival signaling for A549 cells. Moreover, the JNK and p38 phosphorylation in A549 cells was found enhanced in response to OGE treatment, which plays an important role in apoptotic signaling through regulating the activities of pre-existing Bcl-2 family proteins and mediating caspase activation [12].

Recently, several lines of evidence indicate that extracts of *Ocimum* species possess antitumor effects. Ethanolic extract of *Ocimum sanctum* has been reported to induce apoptosis of A549 cell via the intrinsic/mitochondria-dependent pathway [24] and chemical-induced gastric carcinogenesis via inhibition of proliferation, angiogenesis, and apoptosis-associated proteins [25]. Essential oil from *Ocimum sanctum* also has been shown to exert cytotoxic and apoptotic activity to human colorectal adenocarcinoma cell COLO 205 [26]. Moreover, *Ocimum sanctum* combining with *Azadirachta indica* is reported to have synergistic chemopreventive effects on chemical-induced gastric carcinogenesis, which may be attributed to their anti-oxidant, antiangiogenic, antiproliferative, and apoptosis inducing properties [27]. These findings indicate that *Ocimum spp.* extracts possess potent antitumoral activity through inducing apoptosis, inhibiting proliferation and suppressing angiogenesis.

In the present study, we show that OGE activates both intrinsic and extrinsic pathway in A549 cells. Aqueous extract of OG leaf has been reported to inhibit the growth and the migration of breast cancer cell MDA-MB-231 [28]. With the treatment of 0.5% (5 mg/ml) aqueous extract of OG leaf for 72 h, the cell viability of MDA-MB-231 was reduced to approximately 60% of the control. Our results reveal that the lower concentration of OGE (500 and 800 *μ*g/ml) significantly diminishes the cell viability of A549 to 47% and 36% of the control. These findings suggest that lung adenocarcinoma cells are more susceptible to OGE treatment than breast cancer cells.

**Figure 7:**
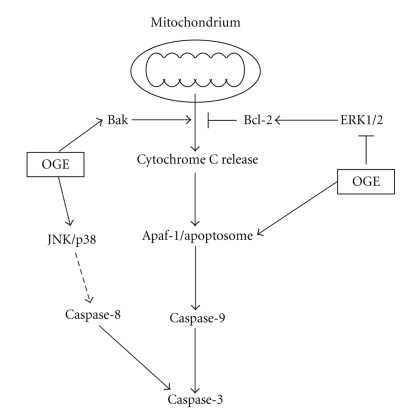
Proposed model for the antitumoral activity of *Ocimum gratissimum* extracts on the human lung adenocarcinoma A549. Our data demonstrated that *Ocimum gratissimum* extract induces activation of caspase-3, caspase-9, and caspase-8, which may attribute to the increase of Apaf-1 and Bak, the decrease of antiapoptotic Bcl-2, as well as the inhibition of ERK1/2 survival signaling and the enhancement of JNK and p38 stress signal cascades.

In conclusion, the present study provides evidences that OGE treatments significantly alter viability of lung adenocarcinoma A549 cells through a synergy of induction of apoptotic signaling and suppression of antiapoptotic signaling, as shown in Figure 7. Moreover, OGE treatment simultaneously inhibits the activation of ERK and enhances the activation of JNK and p38, which is consistent with the enhanced apoptotic signaling and reduced antiapoptotic signaling based on their well-known effects on these signal cascades (Figure 7). By manipulating both arms of apoptotic and antiapoptotic pathway, OGE represents a promisingly effective chemopreventive agent against lung adenocarcinoma.
